# Plasma retinol-binding protein 4 in the first and second trimester and risk of gestational diabetes mellitus in Chinese women: a nested case-control study

**DOI:** 10.1186/s12986-019-0425-9

**Published:** 2020-01-06

**Authors:** Chuyao Jin, Lizi Lin, Na Han, Zhiling Zhao, Zheng Liu, Shusheng Luo, Xiangrong Xu, Jue Liu, Haijun Wang

**Affiliations:** 1 Department of Maternal and Child Health, School of Public Health, Peking University, No. 38 Xueyuan Rd, Haidian District, Beijing, 100191 People’s Republic of China; 2 Maternal and Child Health Hospital of Tongzhou District, Beijing, 101101 China; 3 Department of Epidemiology and Biostatistics, School of Public Health, Peking University, No. 38 Xueyuan Rd, Haidian District, Beijing, 100191 People’s Republic of China

**Keywords:** Gestational diabetes mellitus, Retinol-binding protein 4, Insulin resistance

## Abstract

**Background:**

To assess the association between plasma retinol-binding protein 4 (RBP4) levels both in the first trimester and second trimester and risk of gestational diabetes mellitus (GDM).

**Methods:**

Plasma RBP4 levels and insulin were measured among 135 GDM cases and 135 controls nested within the Peking University Birth Cohort in Tongzhou. Multivariable linear regression analysis was conducted to assess the influence of RBP4 levels on insulin resistance. Conditional logistic regression models were used to compute the odds ratio (OR) and 95% confidence interval (CI) between RBP4 levels and risk of GDM.

**Results:**

The GDM cases had significantly higher levels of RBP4 in the first trimester than controls (medians: 18.0 μg/L vs 14.4 μg/L; *P* < 0.05). Plasma RBP4 concentrations in the first and second trimester were associated with fasting insulin, homeostasis model assessment for insulin resistance (HOMA-IR), and the quantitative insulin sensitivity check index (QUICKI) in the second trimester (all *P* < 0.001). With adjustment for diet, physical activity, and other risk factors for GDM, the risk of GDM increased with every 1-log μg/L increment of RBP4 levels, and the OR (95% CI) was 3.12 (1.08–9.04) for RBP4 in the first trimester and 3.38 (1.03–11.08) for RBP4 in the second trimester.

**Conclusions:**

Plasma RBP4 levels both in the first trimester and second trimester were dose-dependently associated with increased risk of GDM.

## Introduction

Retinol-binding protein 4 (RBP4) was initially identified as a hormone secreted by the liver and acted as a vitamin A transport protein that facilitates the transfer of retinol from the liver to peripheral tissues [1]. However, recent studies revealed that RBP4 could be a new adipokine with potential involvement in the pathogenesis of insulin resistance and type 2 diabetes mellitus (DM) [2–4]. Animal studies showed that injection of RBP4 in normal mice leads to insulin resistance while genetic deletion of *RBP4* gene could enhance insulin sensitivity [2]. In human studies, RBP4 correlates with the magnitude of insulin resistance [3], and elevated RBP4 is associated with an increased risk of type 2 DM [4].

Gestational diabetes mellitus (GDM) develops when insulin secretion from pancreatic beta cells is insufficient to respond to the increased insulin requirements of pregnancy, resulting in hyperglycemia of variable severity [5]. GDM and type 2 DM share similar pathogenetic mechanisms and women with previous GDM have an increased risk of developing type 2 DM in later life [6]. Thus, RBP4 may play a role in the development of insulin resistance in GDM. Given that GDM is associated with a well-documented range of short- and long-term adverse outcomes for the mother and the offspring [5], studying the role of RBP4 for early detection and treatment of GDM is of great importance.

Several previous studies assessed the association between RBP4 and GDM and yielded inconsistent results [7, 8]. A meta-analysis pooled results from 14 case-control studies reported that the serum RBP4 level was associated with the risk of GDM (pooled standardized mean difference = 0.816) [8]. Nevertheless, this meta-analysis only includes one article in the “before 24 weeks” subgroup and no association between serum RBP4 level and GDM risk was found [9]. Recently, a prospective study revealed that early pregnancy RBP4 concentrations were associated with increased risk of GDM [7]. However, none of the previous studies analyzed RBP4 levels at different trimesters before the diagnosis of GDM.

Thus, the current study aimed to investigate the effect of RBP4 levels both in the first trimester and second trimester on insulin resistance and GDM in a prospective cohort of Chinese women, after adjustment for potential confounders including diet and physical activity.

## Material and methods

### Study design

This was a nested case-control study within the Peking University Birth Cohort in Tongzhou (PKUBC-T). The primary aim of the prospective cohort was to investigate the short- and long-term health effects of pre-pregnant and prenatal exposures on mothers and their children. This cohort has been registered in ClinicalTrials.gov (NCT 03814395, see the website for details). Baseline recruitment was conducted between June 2018 and February 2019, and pregnant women who visited the outpatient clinic for the first prenatal examination at Tongzhou maternal and child health hospital and met the following inclusion criteria were recruited: 1) age between 18 to 45 years old; 2) < 14 gestational weeks; 3) resided in Tongzhou during the past half year and have no plan to move out after delivery; 4) plan to have antenatal care and delivery in Tongzhou Maternal and Child Health Hospital. The study was approved by the institutional review boards at Peking University (IRB00001052–18003), and all participants gave written informed consent at the enrollment.

In total, 5477 pregnant women met the inclusion criteria and were recruited at baseline. By February 2019, 3304 women have completed the oral glucose tolerance test (OGTT) and 593 of them were diagnosed with GDM (17.9%). Among 593 women with GDM, 135 cases were randomly selected, and 135 controls were randomly selected among women with normal glucose tolerance (NGT). The characteristics of the selected cases and controls were comparable to all incident GDM cases and non-GDM women, respectively (Additional file 2: Table S1 and Additional file 3: Table S2). Cases and controls were matched for age (±2 years old) and gestation week of taking OGTT by 1:1 ratio. The exclusion criteria included women with history of GDM, family history of diabetes, polycystic ovary syndrome, thyroid disease, cigarette smoking and alcohol consumption.

### Biological sample collection and laboratory measurement

Fasting blood samples were obtained from all participants at the first prenatal visit (< 14 gestational weeks) in the first trimester and during OGTT (24–28 gestational weeks) in the second trimester. Plasma was extracted and measured for fasting plasma glucose (FPG) and biochemical indicators, including triglyceride, total cholesterol, high-density lipoprotein (HDL), low-density lipoprotein (LDL), creatinine, alanine transaminase (ALT), and aspartate aminotransferase (AST) using standard detection methods. Glomerular filtration rate (GFR) was calculated by the modified MDRD equation for Chinese population [10]. Plasma RBP4 and insulin were batch analyzed by the enzyme-linked immunosorbent assay (ELISA) (R&D Systems China, Shanghai), following manufacturer’s instructions. Plasma from the same case-control pair was analyzed on the same plate. All samples are measured in duplicate, and results were averaged.

### Assessment of outcomes

The primary outcomes of interest were insulin resistance and GDM. Homeostasis model assessment (HOMA) was used to estimate insulin resistance [HOMA-IR = insulin/(22.5e^-ln glucose^)] [11]. The quantitative insulin sensitivity check index (QUICKI) was calculated according to the formula: QUICKI = 1/[log(fasting insulin, μU/mL) + log(fasting glucose, mg/dL)] [12]. GDM was diagnosed according to the criteria of the International Association of Diabetic Pregnancy Study Group (IADPSG) guidelines [13]. Women with one or more abnormal values from the 2 h, 75 g OGTT between 24 and 28 gestational weeks will be diagnosed as GDM, with the cut-points of 5.1 mmol/L for fasting, 10.0 mmol/L for 1 h and 8.5 mmol/L for 2 h.

### Assessment of covariates

Covariates were collected at the first prenatal visit, such as maternal characteristics regarding demographic information, pre-pregnancy weight, pregnancy history, smoking status, alcohol intake, family history of diabetes. Dietary intake was recorded for two non-consecutive days and average daily intake of calories was calculated. Physical activity was evaluated using the last 7-day, short form of the International Physical Activity Questionnaire and quantified using metabolic equivalents of task (MET-min week^− 1^) [14]. Each subject’s height, weight, and blood pressure were measured by trained nurses. Weight was routinely measured at subsequent antenatal visits. Body mass index (BMI) was calculated using weight (in kilograms) divided by the square of height (in meters). Gestational weight gain (GWG) before the diagnosis of GDM was calculated as weight measured at or before (within 2 weeks) the diagnosis of GDM minus pre-pregnancy weight.

### Statistical analyses

Continuous variables were presented as medians (interquartile range, IQR) and analyzed for between-group differences using Wilcoxon rank sum test. Categorical variables were analyzed for between-group differences using the χ^2^ test. Multivariable linear regression analysis was conducted to estimate the associations of RBP4 levels in the first and second trimester with fasting insulin, HOMA-IR and QUICKI in the second trimester. Then, study subjects were divided into four groups (Q1, Q2, Q3, and Q4) according to quartiles of RBP4 levels among control group, and the lowest quartile (Q1) was used as the reference group. Conditional logistic regression models were used to compute the odds ratio (OR) and 95% confidence interval (CI) between RBP4 levels and risk of GDM. Three models were established to assess the robustness of the results. In model 1, we adjusted for maternal age, education, occupation, gestational weeks of RBP4 measurements in the first trimester, pre-pregnancy BMI, systolic blood pressure (SBP), and diastolic blood pressure (DBP) at the first prenatal visit, and GWG before OGTT. We additionally adjusted for biochemical indicators involving total cholesterol, triglyceride, HDL, LDL, GFR, ALT, and AST in model 2. Further adjustment for daily intake of calories and weekly physical activity time was conducted in model 3. We also performed tests for linear trend by entering the median value of each RBP4 level as a continuous variable in the models. Restricted cubic spline regressions were used with three knots to examine possible nonlinear relationships between RBP4 levels and risk of GDM. In the sensitivity analysis, we converted all continuous covariates into categorical covariates (classified by median values) to test the robustness of our results. Moreover, we conducted a subgroup analysis to test if maternal age (stratified by median age, < 29 versus ≥29 years) could modify the association between RBP4 and GDM by adding an interaction term to the regression models. Unconditional logistic regression models were applied to compute the results. All analyses were performed with SAS 9.4 and two-sided *P* values < 0.05 was considered to be statistically significant.

## Results

The characteristics of the study subjects are presented in Table 1. No significant differences were found between cases and controls regarding maternal age, education, occupation, gestational weeks at enrollment, pre-pregnancy BMI, GWG before OGTT, weekly physical activity time, daily intake of calories, SBP, DBP, and those biochemical indicators at the first prenatal visit (all *P* > 0.05).

**Table 1:** Baseline characteristics of GDM cases and matched controls

	GDM	Control	*P*
Age, year	29 (28–33)	29 (28–33)	1.00
Education> 12 years, n (%)	110 (81.5)	107 (79.3)	0.55
Employed, n (%)	104 (77.0)	109 (80.7)	0.46
Gweek at enrollment	10 (9–12)	10 (9–12)	0.36
Pre-pregnancy BMI, kg/m^2^	22.2 (20.3–25.1)	22.0 (19.9–24.8)	0.45
GWG before OGTT, kg	8.6 (6.2–11.0)	8.6 (6.4–10.5)	0.56
Weekly PA time, MET-min week^−1^	693 (238–1386)	693 (198–1386)	0.78
Daily intake of calories, kcal/d	1272 (1031–1630)	1242 (936–1683)	0.70
SBP, mmHg	108 (101–116)	110 (102–119)	0.43
DBP, mmHg	67 (62–73)	67 (61–72)	0.66
Total cholesterol, mmol/L	4.0 (3.6–4.4)	3.9 (3.6–4.4)	0.84
Triglyceride, mmol/L	1.2 (0.9–1.4)	1.0 (0.9–1.3)	0.07
HDL cholesterol, mmol/L	1.7 (1.4–1.9)	1.8 (1.5–1.9)	0.26
LDL cholesterol, mmol/L	2.2 (1.9–2.7)	2.3 (1.9–2.6)	0.79
GFR, ml/min/1.73 m^2^	168.6 (151.9–182.6)	172.6 (155.1–191.1)	0.28
ALT, U/L	13 (10–23)	12 (10–19)	0.33
AST, U/L	14 (13–18)	15 (13–17)	0.92
RBP4 in the 1st trimester, μg/L	18.0 (10.2–31.6)	14.4 (9.6–21.8)	0.04
RBP4 in the 2nd trimester, μg/L	18.0 (11.3–29.1)	15.4 (9.6–21.8)	0.08

*Abbreviations*: *GDM* Gestational diabetes mellitus, *Gweek* Gestational week, *BMI* Body mass index, *GWG* Gestational weight gain, *OGTT* Oral glucose tolerance test, *PA* Physical activity, *SBP* Systolic blood pressure, *DBP* Diastolic blood pressure, *HDL* High-density lipoprotein, *LDL* Low-density lipoprotein, *GFR* Glomerular filtration rate, *ALT* Alanine transaminase, *AST* Aspartate aminotransferase, *RBP4* Retinol-binding protein 4

Women with GDM had significantly higher levels of RBP4 in the first trimester than controls (medians: 18.0 μg/L vs 14.4 μg/L; *P* < 0.05; Table 1). Multivariable linear regression analysis showed that plasma RBP4 concentrations in the first and second trimester are the independent factor affecting fasting insulin, HOMA-IR and QUICKI in the second trimester (all *P* < 0.001), after adjustment for potential confounders, including biochemical indicators, daily intake of calories, and physical activity (Table 2).

**Table 2:** Multivariable linear regression analysis for the association of RBP4 in the first trimester and second trimester with fasting insulin, HOMA-IR and QUICKI in the second trimester

	fasting insulin in 2nd trimester		HOMA-IR in 2nd trimester		QUICKI in the 2nd trimester	
	*β*	*P* value	*β*	*P* value	*β*	*P* value
RBP4 in the 1st trimester						
Model 1^a^	1.254	< 0.001	0.275	< 0.001	−2.92 × 10^−3^	< 0.001
Model 2^b^	1.230	< 0.001	0.270	< 0.001	−2.84 × 10^− 3^	< 0.001
Model 3^c^	1.237	< 0.001	0.271	< 0.001	−2.88 × 10^−3^	< 0.001
RBP4 in the 2nd trimester						
Model 1^a^	1.436	< 0.001	0.312	< 0.001	−3.32 × 10^− 3^	< 0.001
Model 2^b^	1.422	< 0.001	0.309	< 0.001	−3.23 × 10^− 3^	< 0.001
Model 3^c^	1.424	< 0.001	0.310	< 0.001	−3.25 × 10^− 3^	< 0.001

*Abbreviations*: *RBP4* Retinol-binding protein 4, *HOMA-IR* Homeostatic model assessment for insulin resistance, *QUICKI* Quantitative insulin sensitivity check index

^a^ Model 1: adjusted for maternal age, education, occupation, gestational weeks of RBP4 measurements in the first trimester, pre-pregnancy BMI, GWG before OGTT, SBP, DBP

^b^ Model 2: Model 1 additional adjusted total cholesterol, triglyceride, HDL, LDL, GFR, ALT, AST

^c^ Model 3: Model 2 additional adjusted daily intake of calories and weekly physical activity time

As shown in Table 3, the highest quartile level of RBP4 had a higher proportion of GDM cases than the lowest quartile (62.7% vs 45.6% in the first trimester, 60.3% vs 47.8% in the second trimester). In multivariable logistic regression analysis, compared with the lowest RBP4 level, subjects in the highest quartile of RBP4 was associated with a significantly higher risk of GDM in both first trimester (aOR = 3.63; 95% CI, 1.30~10.12; *P*-trend = 0.004) and second trimester (aOR = 3.26; 95% CI, 1.09~9.72; *P*-trend = 0.018) after adjusting for maternal age, education, occupation, gestational weeks of RBP4 measurements in the first trimester, pre-pregnancy BMI, GWG before OGTT, SBP, and DBP. After additional adjustment for biochemical indicators, subjects in the highest quartile of RBP4 had 3.72-fold (95% CI, 1.27~10.87; *P*-trend = 0.005) and 3.25-fold (95% CI, 1.02~10.34; *P*-trend = 0.026) higher risk of developing GDM, respectively. In addition, further adjusting for daily intake of calories and physical activity didn’t attenuate this association, and the adjusted OR (95% CI) was 3.71 (1.27~10.84) for RBP4 in the first trimester and 3.30 (1.03~10.52) for RBP4 in the second trimester. We also estimated the risk of GDM associated with every 1-log μg/L increment in RBP4 levels, and the OR (95% CI) in model 3 was 3.12 (1.08~9.04) for RBP4 in the first trimester and 3.38 (1.03~11.08) for RBP4 in the second trimester. Findings of the sensitivity analysis showed that our results were robust after converting all continuous covariates into categorical covariates in the models (results shown in Additional file 4: Table S3). Additional file 1: Figure S1 demonstrated the results of the restricted cubic spline regressions. We found no evidence of a non-linear association between RBP4 levels and the risk of GDM (the *P* value for nonlinearity was 0.47 for the first trimester and 0.41 for the second trimester). In the subgroup analysis, we found that maternal age did not modify the association between RBP4 levels and GDM (*P* for interaction was 0.64 in the first trimester and 0.75 in the second trimester, Additional file 5: Table S4).

**Table 3:** Odds ratio (95% confidence intervals) of GDM associated with different levels of RBP4 in the first trimester and second trimester

	Quartiles of RBP4				*P* for trend	Per 1 log increment
	Q1	Q2	Q3	Q4		
First trimester						
Median (range)	5.8 (4.2–8.2)	12.9 (11.4–14.0)	20.6 (17.7–22.7)	42.1 (32.5–61.6)		
Cases (%)	31 (45.6)	28 (41.8)	34 (50.0)	42 (62.7)		
Model 1^a^	1.00	1.07 (0.44–2.64)	2.06 (0.76–5.58)	3.63 (1.30–10.12)	0.004	3.60 (1.29–10.03)
Model 2^b^	1.00	1.06 (0.41–2.71)	2.58 (0.86–7.75)	3.72 (1.27–10.87)	0.005	3.14 (1.09–9.06)
Model 3^c^	1.00	1.06 (0.41–2.74)	2.60 (0.86–7.86)	3.71 (1.27–10.84)	0.005	3.12 (1.08–9.04)
Second trimester						
Median (range)	5.9 (4.6–8.9)	13.3 (12.0–14.9)	20.1 (17.8–23.7)	40.2 (30.1–54.9)		
Cases (%)	32 (47.8)	27 (39.7)	35 (52.2)	41 (60.3)		
Model 1^a^	1.00	0.89 (0.36–2.17)	2.30 (0.78–6.79)	3.26 (1.09–9.72)	0.018	3.62 (1.17–11.25)
Model 2^b^	1.00	0.88 (0.34–2.25)	2.88 (0.88–9.42)	3.25 (1.02–10.34)	0.026	3.34 (1.03–10.85)
Model 3^c^	1.00	0.89 (0.35–2.30)	2.95 (0.90–9.69)	3.30 (1.03–10.52)	0.028	3.38 (1.03–11.08)

*Abbreviations*: *GDM* Gestational diabetes mellitus, *RBP4* Retinol-binding protein 4

^a^ Model 1: adjusted for maternal age, education, occupation, gestational weeks of RBP4 measurements in the first trimester, pre-pregnancy BMI, GWG before OGTT, SBP, DBP

^b^ Model 2: Model 1 additional adjusted total cholesterol, triglyceride, HDL, LDL, GFR, ALT, AST

^c^ Model 3: Model 2 additional adjusted daily intake of calories and weekly physical activity time

## Discussion

GDM affects around 1 in 7 of pregnancies worldwide [15] and causes serious health risks for the mothers and offspring [5]. Screening for GDM is generally performed between 24 and 28 gestational weeks using an OGTT. If early pregnancy biomarker could be found, it could be a useful tool for early detection and treatment of women at high risk of GDM to improve perinatal and long-term outcomes of GDM women and their offspring. In this prospective study among Chinese women, we found a dose-dependent association between plasma RBP4 levels and GDM risk, which was independent of diet, physical activity, and other risk factors of GDM. Each 1-log μg/L increment in RBP4 level in the first trimester and second trimester was associated with 3.12-fold and 3.38-fold increased risk of GDM respectively, indicating that RBP4 could be a biomarker to identify women at high risk of GDM during early pregnancy.

Mechanism studies found that RBP4 was involved in the pathogenesis of diabetes by inducing insulin resistance through several etiologic pathways. Increasing serum RBP4 could induce the expression of the phosphoenolpyruvate kinase from the liver and impair insulin signaling in muscle [2]. Furthermore, RBP4 may contribute to the development of an inflammatory state in adipose tissue through activating proinflammatory cytokines in macrophages [16]. In our study, we found that RBP4 levels in early pregnancy could predict insulin resistance in the second trimester. Similarly, Su et al. [17] found serum RBP4 measured at the median gestational week 26 in 121 Chinese pregnant women was positively correlated with HOMA-IR (*r* = 0.345, *P* < 0.001). Another study also found RBP4 levels were significantly correlated with HOMA-IR in the first trimester in healthy pregnant women (*r* = 0.475, *P* < 0.001) [18]. However, a prospective study including 172 women with GDM and 361 non-GDM Thai women suggested the RBP4 level at the time of OGTT was not associated with insulin resistance [19]. The conflicting results may in part be due to differences in the study populations, measuring methods, and different gestational weeks for RBP4 measurements. By contrast, a randomized and double-blind trial revealed that after 16 weeks of sitagliptin administration, serum levels of RBP4 were significantly decreased in the treatment group, which was positively correlated with improved insulin resistance parameters [20]. Further research is needed to understand the underlying mechanism between RBP4 and insulin resistance during pregnancy.

The relationship between RBP4 levels in early pregnancy and the risk of GDM have been assessed by limited studies and inconclusive results were yielded. A prospective study found that compared with the first quartile of RBP4 in the first trimester, adjusted ORs of GDM for the second, third and fourth quartile were 1.54, 3.05 and 6.36 respectively [7]. Another case-control study found participants in the highest quartile of serum RBP4 had a 1.89 times higher risk of GDM compared with participants in the lowest quartile, while the relationship did not reach statistical significance after adjustment for maternal age, race, family history of diabetes, and pre-pregnancy overweight status [21]. However, Fruscalzo et al. [9] found the concentrations of RBP4 in 11 insulin-treated GDM cases were significantly lower than 44 controls. Another research revealed an insignificant difference in serum concentrations of RBP4 between women who developed GDM and controls [22]. Previous studies were mainly limited by testing blood levels of RBP4 only once before the diagnosis of GDM and small sample size. In our study, we found RBP4 levels both in the first trimester and second trimester were associated with GDM risk, and a dose-dependent association was also revealed.

Several limitations of the current study should be considered. First, although we adjusted for several potential confounders such as diet and physical activity, we cannot exclude the possibility of unmeasured confounders. Second, RBP4 is generally present in free and transthyretin-bound forms for the transport of retinol. Previous study reported that the RBP4:retinol ratio and the RBP4:transthyretin ratio might be more informative than RBP4 levels alone when assessing insulin resistance during pregnancy [23]. We did not measure retinol and transthyretin in this study, thus we can’t compare the function between free and bound RBP4. Third, the sample size of this study was relatively small.

## Conclusions

In summary, we reported for the first time a dose-dependent association between RBP4 levels both in the first trimester and second trimester and GDM risk. In addition, plasma RBP4 in the first and second trimester is associated with insulin resistance in the second trimester. Further research is warranted to determine the underlying mechanism and examine if a reduction in RBP4 concentrations could lower the risk of GDM.

## Supplementary information

**Additional file 1: Figure S1.** The dose-response analysis between GDM and RBP4 levels in the first trimester (A) and the second trimester (B) with restricted cubic spline. Conditional logistic regression models were adjusted for maternal age, education, occupation, gestational weeks of RBP4 measurements in the first trimester, pre-pregnancy BMI, GWG before OGTT, SBP, DBP, total cholesterol, triglyceride, HDL, LDL, GFR, ALT, AST, daily intake of calories, and weekly physical activity time. Bold lines are ORs and dashed lines are 95% CIs. The horizontal line is the reference line. Abbreviation: GDM, gestational diabetes mellitus; RBP4, retinol-binding protein 4.

**Additional file 2: Table S1.** Comparison of the baseline characteristics between GDM cases included in the analysis and all GDM cases. Abbreviation: GDM, gestational diabetes mellitus; Gweek, gestational week; BMI, body mass index; GWG, gestational weight gain; OGTT, oral glucose tolerance test; PA, physical activity; SBP, systolic blood pressure; DBP, diastolic blood pressure; HDL, high-density lipoprotein; LDL, low-density lipoprotein; GFR, glomerular filtration rate; ALT, alanine transaminase; AST, aspartate aminotransferase.

**Additional file 3: Table S2.** Comparison of the baseline characteristics between controls included in the analysis and all controls. Abbreviation: GDM, gestational diabetes mellitus; Gweek, gestational week; BMI, body mass index; GWG, gestational weight gain; OGTT, oral glucose tolerance test; PA, physical activity; SBP, systolic blood pressure; DBP, diastolic blood pressure; HDL, high-density lipoprotein; LDL, low-density lipoprotein; GFR, glomerular filtration rate; ALT, alanine transaminase; AST, aspartate aminotransferase.

**Additional file 4: Table S3.** Odds ratio (95% confidence intervals) of GDM associated with different levels of RBP4 in the first trimester and second trimester after converting all continuous covariates into categorical covariates in the models. Models were adjusted for maternal age (≤29, > 29 years), education (≤12, > 12 years), occupation (employed, unemployed), gestational weeks of RBP4 measurements in the first trimester (≤10, > 10 weeks), pre-pregnancy BMI (≤22, > 22 kg/m^2^), GWG before OGTT (≤8.7, > 8.7 kg), SBP (≤110, > 110 mmHg), DBP (≤67, > 67 mmHg), total cholesterol (≤4.0, > 4.0 mmol/L), triglyceride (≤1.1, > 1.1 mmol/L), HDL (≤1.7, > 1.7 mmol/L), LDL (≤2.3, > 2.3 mmol/L), GFR (≤169.7, > 169.7 ml/min/1.73 m^2^), ALT (≤13, > 13 U/L), AST (≤14, > 14 U/L), daily intake of calories (≤1254, > 1254 kcal/d) and weekly physical activity time (≤693, > 693 MET-min week^− 1^). Abbreviation: GDM, gestational diabetes mellitus; RBP4, retinol-binding protein 4.

**Additional file 5: Table S4.** Odds ratio (95% confidence intervals) of GDM associated with different levels of RBP4 in the first trimester and second trimester stratified by maternal age. Unconditional logistic regression models were adjusted for maternal age, education, occupation, gestational weeks of RBP4 measurements in the first trimester, pre-pregnancy BMI, GWG before OGTT, SBP, DBP, total cholesterol, triglyceride, HDL, LDL, GFR, ALT, AST, daily intake of calories, and weekly physical activity time. Abbreviation: GDM, gestational diabetes mellitus; RBP4, retinol-binding protein 4.

## Data Availability

The datasets used and/or analysed during the current study are available from the corresponding author on reasonable request.

## Abbreviations

ALT: Alanine transaminase
AST: Aspartate aminotransferase
BMI: Body mass index
DBP: Diastolic blood pressure
DM: Diabetes mellitus
ELISA: Enzyme-linked immunosorbent assay
FPG: Fasting plasma glucose
GDM: Gestational diabetes mellitus
GFR: Glomerular filtration rate
GWG: Gestational weight gain
HDL: High-density lipoprotein
HOMA: Homeostasis model assessment
HOMA-IR: Homeostasis model assessment for insulin resistance
IADPSG: International Association of Diabetic Pregnancy Study Group
LDL: Low-density lipoprotein
NGT: Normal glucose tolerance
OGTT: Oral glucose tolerance test
PKUBC-T: Peking University Birth Cohort in Tongzhou
QUICKI: Quantitative insulin sensitivity check index
RBP4: Retinol-binding protein 4
SBP: Systolic blood pressure
