# Supplementary figures and images for: Plasma retinol-binding protein 4 in the first and second trimester and risk of gestational diabetes mellitus in Chinese women: a nested case-control study
Source: Nutr Metab (Lond). 2020 Jan 6;17:1. doi: 10.1186/s12986-019-0425-9 (PMC6945716; doi:10.1186/s12986-019-0425-9)

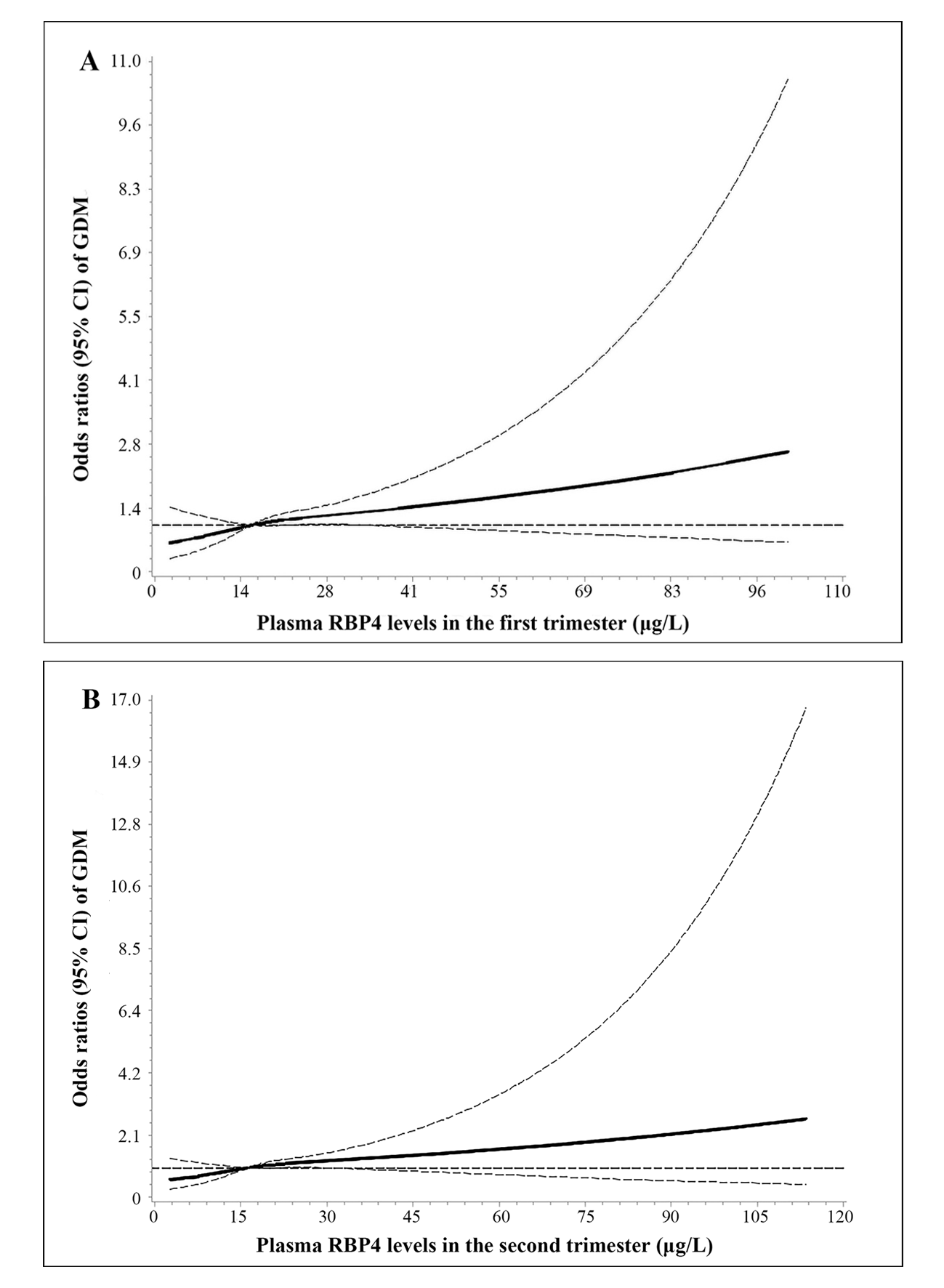

Supplement: Supplementary file 1 — Additional file 1: Figure S1. The dose-response analysis between GDM and RBP4 levels in the first trimester (A) and the second trimester (B) with restricted cubic spline. Conditional logistic regression models were adjusted for maternal age, education, occupation, gestational weeks of RBP4 measurements in the first trimester, pre-pregnancy BMI, GWG before OGTT, SBP, DBP, total cholesterol, triglyceride, HDL, LDL, GFR, ALT, AST, daily intake of calories, and weekly physical activity time. Bold lines are ORs and dashed lines are 95% CIs. The horizontal line is the reference line. Abbreviation: GDM, gestational diabetes mellitus; RBP4, retinol-binding protein 4. [file 12986_2019_425_MOESM1_ESM.tif]
