# Supplementary material for: Plasma retinol-binding protein 4 in the first and second trimester and risk of gestational diabetes mellitus in Chinese women: a nested case-control study
Source: Nutr Metab (Lond). 2020 Jan 6;17:1. doi: 10.1186/s12986-019-0425-9 (PMC6945716; doi:10.1186/s12986-019-0425-9)
Supplement: Supplementary file 2 — Additional file 2: Table S1. Comparison of the baseline characteristics between GDM cases included in the analysis and all GDM cases. Abbreviation: GDM, gestational diabetes mellitus; Gweek, gestational week; BMI, body mass index; GWG, gestational weight gain; OGTT, oral glucose tolerance test; PA, physical activity; SBP, systolic blood pressure; DBP, diastolic blood pressure; HDL, high-density lipoprotein; LDL, low-density lipoprotein; GFR, glomerular filtration rate; ALT, alanine transaminase; AST, aspartate aminotransferase. [file 12986_2019_425_MOESM2_ESM.docx]

**Table S1** Comparison of the baseline characteristics between GDM cases included in the analysis and all GDM cases

|  | GDM cases included in the analysis  (n=135) | All GDM cases  (n=593) | *P* |
| --- | --- | --- | --- |
| Age, year | 29 (28-33) | 30 (28-33) | 0.68 |
| Education>12 years, n (%) | 110 (81.5) | 479 (80.8) | 0.43 |
| Employed, n (%) | 104 (77.0) | 466 (78.6) | 0.16 |
| Gweek at enrollment | 10 (9-12) | 10 (9-12) | 0.41 |
| Pre-pregnancy BMI, kg/m^2^ | 22.2 (20.3-25.1) | 23.2 (20.8-25.4) | 0.06 |
| GWG before OGTT, kg | 8.6 (6.2-11.0) | 8.5 (6.0-11.1) | 0.55 |
| Weekly PA time, MET-min week^-1^ | 693 (238-1386) | 660 (198-1188) | 0.56 |
| Daily intake of calories, kcal/d | 1272 (1031-1630) | 1294 (1014-1638) | 0.77 |
| SBP, mmHg | 108 (101-116) | 110 (103-119) | 0.16 |
| DBP, mmHg | 67 (62-73) | 67 (61-73) | 0.95 |
| Total cholesterol, mmol/L | 4.0 (3.6-4.4) | 4.0 (3.6-4.4) | 0.67 |
| Triglyceride, mmol/L | 1.2 (0.9-1.4) | 1.2 (0.9-1.5) | 0.21 |
| HDL cholesterol, mmol/L | 1.7 (1.4-1.9) | 1.6 (1.4-1.9) | 0.39 |
| LDL cholesterol, mmol/L | 2.2 (1.9-2.7) | 2.3 (1.9-2.7) | 0.39 |
| GFR, ml/min/1.73 m^2^ | 168.6 (151.9-182.6) | 166.0 (150.1-186.0) | 0.82 |
| ALT, U/L | 13 (10-23) | 14 (10-21) | 0.97 |
| AST, U/L | 14 (13-18) | 14 (13-17) | 0.43 |

Abbreviation: GDM, gestational diabetes mellitus; Gweek, gestational week; BMI, body mass index; GWG, gestational weight gain; OGTT, oral glucose tolerance test; PA, physical activity; SBP, systolic blood pressure; DBP, diastolic blood pressure; HDL, high-density lipoprotein; LDL, low-density lipoprotein; GFR, glomerular filtration rate; ALT, alanine transaminase; AST, aspartate aminotransferase.
