# Supplementary material for: Plasma retinol-binding protein 4 in the first and second trimester and risk of gestational diabetes mellitus in Chinese women: a nested case-control study
Source: Nutr Metab (Lond). 2020 Jan 6;17:1. doi: 10.1186/s12986-019-0425-9 (PMC6945716; doi:10.1186/s12986-019-0425-9)
Supplement: Supplementary file 3 — Additional file 3: Table S2. Comparison of the baseline characteristics between controls included in the analysis and all controls. Abbreviation: GDM, gestational diabetes mellitus; Gweek, gestational week; BMI, body mass index; GWG, gestational weight gain; OGTT, oral glucose tolerance test; PA, physical activity; SBP, systolic blood pressure; DBP, diastolic blood pressure; HDL, high-density lipoprotein; LDL, low-density lipoprotein; GFR, glomerular filtration rate; ALT, alanine transaminase; AST, aspartate aminotransferase. [file 12986_2019_425_MOESM3_ESM.docx]

**Table S2** Comparison of the baseline characteristics between controls included in the analysis and all controls

|  | Controls included in the analysis  (n=135) | All controls  (n=2,711) | *P* |
| --- | --- | --- | --- |
| Age, year | 29 (28-33) | 29 (27-32) | 0.01 |
| Education>12 years, n (%) | 107 (79.3) | 2702 (80.0) | 0.97 |
| Employed, n (%) | 109 (80.7) | 2182 (80.5) | 0.99 |
| Gweek at enrollment | 10 (9-12) | 10 (9-12) | 0.24 |
| Pre-pregnancy BMI, kg/m^2^ | 22.0 (19.9-24.8) | 21.5 (19.8-23.8) | 0.15 |
| GWG before OGTT, kg | 8.6 (6.4-10.5) | 8.2 (6.2-10.3) | 0.41 |
| Weekly PA time, MET-min week^-1^ | 693 (198-1386) | 693 (198-2386) | 0.61 |
| Daily intake of calories, kcal/d | 1242 (936-1683) | 1302 (1014-1679) | 0.32 |
| SBP, mmHg | 110 (102-119) | 109 (102-117) | 0.68 |
| DBP, mmHg | 67 (61-72) | 66 (60-72) | 0.59 |
| Total cholesterol, mmol/L | 3.9 (3.6-4.4) | 3.9 (3.5-4.4) | 0.33 |
| Triglyceride, mmol/L | 1.0 (0.9-1.3) | 1.0 (0.8-1.3) | 0.69 |
| HDL cholesterol, mmol/L | 1.8 (1.5-1.9) | 1.7 (1.5-1.9) | 0.69 |
| LDL cholesterol, mmol/L | 2.3 (1.9-2.6) | 2.2 (1.9-2.6) | 0.24 |
| GFR, ml/min/1.73 m^2^ | 172.6 (155.1-191.1) | 169.5 (153.9-187.2) | 0.56 |
| ALT, U/L | 12 (10-19) | 12 (10-18) | 0.22 |
| AST, U/L | 15 (13-17) | 14 (13-17) | 0.51 |

Abbreviation: GDM, gestational diabetes mellitus; Gweek, gestational week; BMI, body mass index; GWG, gestational weight gain; OGTT, oral glucose tolerance test; PA, physical activity; SBP, systolic blood pressure; DBP, diastolic blood pressure; HDL, high-density lipoprotein; LDL, low-density lipoprotein; GFR, glomerular filtration rate; ALT, alanine transaminase; AST, aspartate aminotransferase.
