# Supplementary material for: Plasma retinol-binding protein 4 in the first and second trimester and risk of gestational diabetes mellitus in Chinese women: a nested case-control study
Source: Nutr Metab (Lond). 2020 Jan 6;17:1. doi: 10.1186/s12986-019-0425-9 (PMC6945716; doi:10.1186/s12986-019-0425-9)
Supplement: Supplementary file 4 — Additional file 4: Table S3. Odds ratio (95% confidence intervals) of GDM associated with different levels of RBP4 in the first trimester and second trimester after converting all continuous covariates into categorical covariates in the models. Models were adjusted for maternal age (≤29, > 29 years), education (≤12, > 12 years), occupation (employed, unemployed), gestational weeks of RBP4 measurements in the first trimester (≤10, > 10 weeks), pre-pregnancy BMI (≤22, > 22 kg/m2), GWG before OGTT (≤8.7, > 8.7 kg), SBP (≤110, > 110 mmHg), DBP (≤67, > 67 mmHg), total cholesterol (≤4.0, > 4.0 mmol/L), triglyceride (≤1.1, > 1.1 mmol/L), HDL (≤1.7, > 1.7 mmol/L), LDL (≤2.3, > 2.3 mmol/L), GFR (≤169.7, > 169.7 ml/min/1.73 m2), ALT (≤13, > 13 U/L), AST (≤14, > 14 U/L), daily intake of calories (≤1254, > 1254 kcal/d) and weekly physical activity time (≤693, > 693 MET-min week− 1). Abbreviation: GDM, gestational diabetes mellitus; RBP4, retinol-binding protein 4. [file 12986_2019_425_MOESM4_ESM.docx]

**Table S3** Odds ratio (95% confidence intervals) of GDM associated with different levels of RBP4 in the first trimester and second trimester after converting all continuous covariates into categorical covariates in the models

|  | *OR (95% CI)* | *P* value | *P* for trend |
| --- | --- | --- | --- |
| Quartiles of RBP4 in the 1^st^ trimester |  |  |  |
| Q1 | 1.00 | - | 0.008 |
| Q2 | 0.89 (0.35-2.30) | 0.814 |  |
| Q3 | 1.96 (0.70-5.47) | 0.200 |  |
| Q4 | 3.32 (1.16-9.48) | 0.025 |  |
| Per 1 log increment in the 1^st^ trimester | 3.48 (1.20-10.13) | 0.022 |  |
| Quartiles of RBP4 in the 2^nd^ trimester |  |  |  |
| Q1 | 1.00 | - | 0.010 |
| Q2 | 1.09 (0.42-2.85) | 0.855 |  |
| Q3 | 2.63 (0.83-8.32) | 0.099 |  |
| Q4 | 4.13 (1.30-13.15) | 0.016 |  |
| Per 1 log increment in the 2^nd^ trimester | 4.19 (1.27-13.84) | 0.019 |  |

Models were adjusted for maternal age (≤29, >29 years) , education (≤12, >12 years), occupation (employed, unemployed), gestational weeks of RBP4 measurements in the first trimester (≤10, >10 weeks), pre-pregnancy BMI (≤22, >22 kg/m^2^), GWG before OGTT (≤8.7, >8.7 kg), SBP (≤110, >110 mmHg), DBP (≤67, >67 mmHg), total cholesterol (≤4.0, >4.0 mmol/L), triglyceride (≤1.1, >1.1 mmol/L), HDL (≤1.7, >1.7 mmol/L), LDL (≤2.3, >2.3 mmol/L), GFR (≤169.7, >169.7 ml/min/1.73 m^2^), ALT (≤13, >13 U/L), AST (≤14, >14 U/L), daily intake of calories (≤1254, >1254 kcal/d) and weekly physical activity time (≤693, >693 MET-min week^-1^).

Abbreviation: GDM, gestational diabetes mellitus; RBP4, retinol-binding protein 4.
