# Supplementary material for: Plasma retinol-binding protein 4 in the first and second trimester and risk of gestational diabetes mellitus in Chinese women: a nested case-control study
Source: Nutr Metab (Lond). 2020 Jan 6;17:1. doi: 10.1186/s12986-019-0425-9 (PMC6945716; doi:10.1186/s12986-019-0425-9)
Supplement: Supplementary file 5 — Additional file 5: Table S4. Odds ratio (95% confidence intervals) of GDM associated with different levels of RBP4 in the first trimester and second trimester stratified by maternal age. Unconditional logistic regression models were adjusted for maternal age, education, occupation, gestational weeks of RBP4 measurements in the first trimester, pre-pregnancy BMI, GWG before OGTT, SBP, DBP, total cholesterol, triglyceride, HDL, LDL, GFR, ALT, AST, daily intake of calories, and weekly physical activity time. Abbreviation: GDM, gestational diabetes mellitus; RBP4, retinol-binding protein 4. [file 12986_2019_425_MOESM5_ESM.docx]

**Table S4** Odds ratio (95% confidence intervals) of GDM associated with different levels of RBP4 in the first trimester and second trimester stratified by maternal age

|  |  | Quartiles of RBP4 | | | | *P* for trend | *P* for interaction |
| --- | --- | --- | --- | --- | --- | --- | --- |
|  | n | Q1 | Q2 | Q3 | Q4 |  |  |
| First trimester |  |  |  |  |  |  |  |
| Age<29 years | 136 | 1 | 0.87 (0.28-2.71) | 1.22 (0.38-3.86) | 1.32 (0.42-4.13) | 0.53 | 0.64 |
| Age≥29 years | 134 | 1 | 0.75 (0.25-2.25) | 2.24 (0.74-6.73) | 3.29 (1.03-10.54) | 0.01 |  |
| Second trimester |  |  |  |  |  |  |  |
| Age<29 years | 136 | 1 | 0.69 (0.23-2.05) | 1.10 (0.35-3.45) | 1.42 (0.46-4.38) | 0.48 | 0.75 |
| Age≥29 years | 134 | 1 | 0.83 (0.26-2.60) | 1.95 (0.62-6.12) | 2.11 (0.68-6.56) | 0.11 |  |

Unconditional logistic regression models were adjusted for the same set of covariates for model 3 in Table 3.

Abbreviation: GDM, gestational diabetes mellitus; RBP4, retinol-binding protein 4.
